# Salt Stress Signals on Demand: Cellular Events in the Right Context

**DOI:** 10.3390/ijms21113918

**Published:** 2020-05-30

**Authors:** Ahmed Ismail, Islam El-Sharkawy, Sherif Sherif

**Affiliations:** 1 Department of Horticulture, Faculty of Agriculture, Damanhour University, P.O. Box 22516, Damanhour, Egypt; ahmed.ismail@damanhour.edu.eg; 2 Florida A&M University, Center for Viticulture and Small Fruit Research. 6361 Mahan Drive, Tallahassee, FL 32308, USA; islam.elsharkawy@famu.edu; 3 Alson H. Smith Jr. Agricultural Research and Extension Center, School of Plant and Environmental Sciences, Virginia Tech, Winchester, VA 22062, USA

**Keywords:** salinity, osmotic and ionic stress, salt-related signals, adaptive cellular responses, aquaporins, NSCCs, GIPC, FERONIA, Ca^2+^, H^+^, ROS

## Abstract

Plant stress is a real dilemma; it puzzles plant biologists and is a global problem that negatively affects people’s daily lives. Of particular interest is salinity, because it represents one of the major water-related stress types. We aimed to determine the signals that guide the cellular-related events where various adaptation mechanisms cross-talk to cope with salinity-related water stress in plants. In an attempt to unravel these mechanisms and introduce cellular events in the right context, we expansively discussed how salt-related signals are sensed, with particular emphasis on aquaporins, nonselective cation channels (NSCCs), and glycosyl inositol phosphorylceramide (GIPC). We also elaborated on the critical role Ca2^+^, H^+^, and ROS in mediating signal transduction pathways associated with the response and tolerance to salt stress. In addition, the fragmentary results from the literature were compiled to develop a harmonized, informational, and contemplative model that is intended to improve our perception of these adaptative mechanisms and set a common platform for plant biologists to identify intriguing research questions in this area.

## 1. Review Scope: Signaling Signatures Shape the Consequences

Unlike animals that can exhibit the fight or flight response, plants are sessile organisms that have only one approach when encountering stress—adaptation. Plants constantly monitor an ever-changing environment, sense the incoming signals, and then correctly integrate and decode the signals to formulate a battery of signaling molecules that ultimately enable them to cope with stress. Water-related stress (e.g., drought and salinity) represents one of the major types of plant stressors, of which osmotic stress is considered a general component [1]. In the following sections, we focus on salinity, along with its ionic and osmotic components. We elaborate on the main elements of the term “signals on demand” by answering two questions: (1) how do plants sense salt-related signals? and (2) how are these signals correctly decoded and processed to formulate adaptive cellular responses? 

## 2. Sensing the Upcoming Signals

### 2.1. Aquaporin Water Channels: Debatable Osmosensors

**Figure 1:**
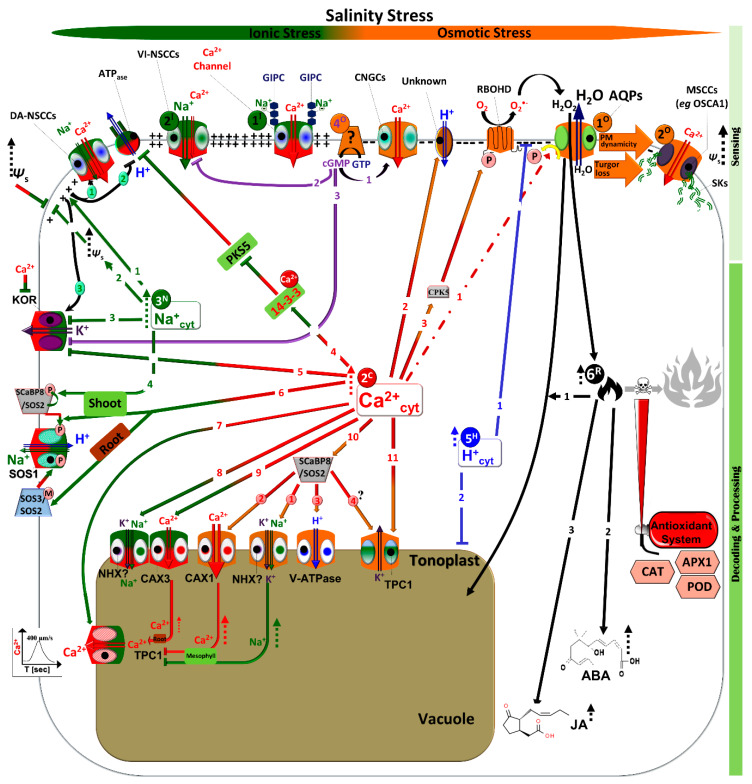
Molecular events and respective responsive cellular organelles/compartments under salt- and osmotic-related stress signaling. Half dark green-half red colored bars represent ionic-related stress and the orange color refers to osmotic stress as a common water-related stress component. Under different water-related stress types, fluxes of Na^+^, Ca^2+^, H^+^, and ROS take place with different spatiotemporal signatures, channeling plant cells to either adaptation or cell death. Details are given in the discussion. Green-colored arrows indicate specificity to ionic events (3^N^), while half green-half red colored arrows indicate Ca^2+^-activated signals (2^C^) that are related to ion stress. On the other hand, Ca^2+^-activated signals (2^C^) under osmotic stress are represented by red-orange colored arrows. Tow-line colored arrows indicate activation of ion fluxes on the plasma membrane and tonoplast by salinity/osmotic stress, where tow-line arrows colored with green, red, and blue represent Na^+^, Ca^2+^, H^+^, and K^+^ flux, while tow-lines with dark blue colored arrows represent the H_2_O flux. Dashed arrows refer to a significant induction under stress. Green, red, blue, and black dashed arrows indicate activation of Ca^2+^, H^+^, and ROS signaling adaptation pathways, respectively, under water-related stress. Abbreviations: ROS: reactive oxygen species; *Ψs* osmotic potential; KOR: K^+^ outward rectifiers; NSCCs: nonselective cation channels; SOS: salt overly sensitive; NHX1: vacuolar Na^+^/H^+^ exchanger 1; TPC1: two pore channel 1 ; GIPC: Glycosyl Inositol Phospho Ceramides; ABA: abscisic acid; JA: jasmonic acid; CAT: catalase; POD: peroxidase; GST: Glutathione S-transferase; AQPs: aquaporins; CAXs: Ca^2+/^H^+^ exchangers; VI-NSCCs: voltage-insensitive- NSCCs; DA-NSCCs: depolarization-activated-NSCCs; SCaBP8: SOS3-like calcium binding protein 8; PKS5: SOS2-like protein kinase; V-ATPases: V-type H^+^-ATPases; OSCAs: reduced hyperosmolality-induced [Ca^2+^]i increase; MSCCs: mechanosensitive NSCCs; CNGCs: cyclic nucleotide gated NSCCs; cAMP: cyclic adenosine monophosphate; cGMP: cyclic guanosine monophosphate; RBOHD: respiratory burst oxidase homolog D; CPK5: calcium-dependent protein kinase 5.

When it comes to plant–water relations, aquaporins (AQPs) play a central role in transport and adaptation processes. AQPs are intrinsic membrane proteins that channel not only H_2_O, but also a high diversity of small and noncharged substrates, such as H_2_O_2_, CO_2_, and urea [2,3]. Based on their cellular localization and functions, AQPs can be subdivided into at least four subfamilies, including the plasma membrane intrinsic proteins (PIPs) and the tonoplast intrinsic proteins (TIPs) [4]. Under water-related stress, the reduction in plant hydraulic conductivity is a common response mediated by AQPs, which have been suggested to act as osmosensors [5]. AQP gating depends on apoplastic/cellular water potential, but occurs at the cytoplasmic side by de/phosphorylation (and/or protonation, in the case of cellular acidosis), which, along with other downregulation processes (e.g., ubiquitination and internalization), have been reported under water-related stress states [6,7,8]. For example, NaCl treatment decreases phosphorylation of AtPIP2;1 and AtPIP2;2, which are among the most abundant AQPs in Arabidopsis roots [9], as well as AtPIP3, AtPIP2;5 AtPIP2;7 [10,11]. Further investigation using AtPIP2;1 showed that the Ser^283^ residue, which is required for guiding AtPIP2;1 to the plasma membrane, is the dephosphorylation site targeted by NaCl stress; hence, nonphosphorylated AtPIP2;1 intracellularly accumulates under increased salinity [12]. The inversion of water flux through the AQP aqueous filter (notably PIPs) under osmotic imbalance may represent the earliest osmosensing action (Figure 1 [1^O^]). In contrast to a steady-state condition, water-related stress raises extracellular osmolarity in conjunction with low apoplastic H_2_O potential. Therefore, an efflux of water out of the cell will dominate the influx process through AQP aqueous filters, leading to cytosolic osmotic disturbances and the loss of cell turgor pressure [13]. These intracellular changes, in turn, might be sensed by the plasma membrane (PM) itself and/or the cell cytoskeleton (mainly microtubules), which subsequently activate the mechanosensitive cation “Ca^2+^” channels (MSCCs) (Figure 1 [2^O^]) [14]. The open MSCCs increase the Ca^2+^ influx [15], which in turn can result in the gating of AQPs, either directly (per se) or indirectly, to prevent further water loss until osmotic readjustment is achieved (Figure 1 [2^C→1^]).

On the contrary, the role of AQPs in stomatal behavior is pivotal for the regulation of the plant’s water status. For example, increasing the water activity (water efflux) of the guard cell via AQPs is a prerequisite for stomatal closure under water stress [4]. Unfortunately, owing to overlapping function of the PIP homologs, a direct link is still missing. Recently, Grondin et al. [16] showed that open stomata 1 (OST1; also known as Snf1-related protein kinase 2.6; SnRK2.6) can phosphorylate PIP2;1 at Ser^121^ in an ABA-dependent manner. Furthermore, transgenic Arabidopsis *pip2*;*1-1* and *pip2*;*1-2* mutants exhibit ABA-specific defects in their stomatal movement [16]. However, by using the same mutants, Wang et al. [17] found that the PIP2;1 mutation alone was insufficient to impair ABA, as well as CO_2_, and that the regulation of stomatal movements in the latter response was comparable to that in *pip2*;*1* mutant and wild type leaves. In drought-stressed *Vitis vinifera*, stomatal closure occurs before the significant increase in foliar ABA. However, ABA induction under water-related stress activates two types of anion channels in a Ca^2+^-dependent manner, subsequently depolarizing the guard cell membranes [18]. The latter consequence inhibits H^+^-ATPase pump activation, resulting in the bioenergetic loss of membranes, activating K^+^ outward rectifiers (KORs), and thereby causing stomatal closure [19]. Interestingly, the involvement of ABA in stomatal regulation was shown to be absent in the early-diverging vascular plant lineages (lycophytes and ferns), and active stomatal control has evolved in seed plants to enhance water regulation and adaptation [20]. In light of these data, stomatal closure is suggested to be induced by passive hydraulic signals, but is maintained by ABA [21]. This might be another evidence of AQPs operating as osmosensors; however, unequivocal proof is still needed.

### 2.2. MSCCs Translate Mechanical Stress into the Ca^2+^ Signal

Nonselective cation channels (NSCCs) are a diverse group of integral membrane proteins that passively flux cations across the PM and other cellular endomembranes to their electrochemical gradients, which are tightly controlled by gating. Although the NSCC classification is problematic, they play a critical role in plant signaling and development, including during stress responses [22]. MSCCs, a class of mechanosensors, directly transduce mechanical stimuli (e.g., osmotic pressure) into the Ca^2+^ influx and are of particular interest.

For instance, the PM-localized two stretch-activated Ca^2+^-permeable MCA (Mid1-Complementing Activity) channels mediate osmotic stress responses and Ca^2+^ homeostasis in plants [15]. Although both MCA1 and MCA2 mediate PM Ca^2+^, they have overlapping and distinct spatial expression patterns and functions, as *mca1* and *mca2* null mutant roots exhibit root deficiency while penetrating into hard agar and taking up Ca^2+^, respectively [23,24]. Interestingly, the *mca1*;*mca2* double mutant, which has a compromised phenotype, does not show any significant Ca^2+^ alternation under NaCl stress compared to the wild type [25]. In the same line, the PM mechanosensitive channel of small conductance (MscS)-like family *msl4,5,6,9,10* quintuple mutant showed the same Ca^2+^ induction pattern [25], and no distinct phenotype was observed when challenged by a variety of stressors, including osmotic, NaCl, and dehydration stressors [26]. Therefore, other subclasses of MSCCs might act in concert with AQPs—and maybe with PM per se and/or the cell cytoskeleton—to handle common water-related osmotic stress (Figure 1 [2^O^]), thereby enhancing the Ca^2+^ influx (Figure 1 [2^C→1^]). Of particular interest are the newly identified MSCCs termed the OSCAs (reduced hyperosmolality-induced [Ca^2+^]i increase), in which the osmotically induced Ca^2+^ signaling is impaired in the guard cells and root cells of the knock-down osca1 mutant [27]. Therefore, OSCA1 is suggested to act as an osmosensor [27,28]. The emerging role of OSCAs and/or other MSCCs may help elucidate Ca^2+^ signaling under water-related stress [29].

One intriguing question is by which mechanism do these MSCCs open and close? Hamilton et al. [15] reviewed two speculated mechanisms. In the context of the mechanical force derived by water-related stress, another essential query involves the possibility that other cellular components are involved, such as the cytoskeleton, the cell wall (exoskeleton), and the PM–cell wall anchors, known as receptor-like kinases (RLKs) [30]. The dependency of AQP PIP activity on water potential differences highlights the importance of the cytoskeleton—in combination with PM dynamicity—in gating MSCCs, and hence activating the downstream Ca^2+^ signaling cascade (Figure 1 [1^O^ and 2^O^]). Indeed, hypo- and hyperosmotic challenges promote cytoskeletal reorganization (actin “AFs” or microtubules “MTs”), which in turn results in modifying cell hydraulic conductivity [31,32,33]. In addition, the MTs-induced MSCC activity upstream of the Ca^2+^ influx is further supported, since the sole application of either a Ca^2+^ blocker (GdCl_3_) or a Ca^2+^ chelator (EGTA) does not affect the *Lp* value [30]. Notably, the cytoskeletal components are not only involved in cell signaling, but also in plant vesicle endocytosis and exocytosis [34]. Moreover, MTs are essential for plant cell wall formation by guiding cellulose synthase enzymes to the PM, the mechanism that is inhibited by salinity-induced MTs depolarization [35]. In contrast, two companion of cellulose synthase (CC1 and CC2) proteins promote MTs assembly, restarting cellulose synthesizing machinery and improving salt adaptation [36]. Recently, the CC1 was found to bind to MTs via its N-terminal hydrophobic domains [37]. Future research on AQPs, cytoskeleton, RLKs, and MSCCs and their levels of sensing the osmotic signal, may unravel these aspects. 

### 2.3. GIPC Sphingolipids: Ionic (Na^+^) Sensors

Under increased salinity, and in contrast to other water stress types, the increase in water and soil ions (mainly NaCl) leads to salt-specific ionic stress that can disrupt cell wall integrity. Indeed, Na^+^ can displace pectin-bound Ca^2+^ and hence interrupt pectin cross-linking in vitro [38,39]. Interestingly, the malectin-like-domain RK FERONIA (FER) can physically interact with pectin via its extracellular domain, and in combination with its co-receptor glycosylphosphatidylinositol-anchored protein (GPI-AP) LORELEI-like GPI-AP1 (LLG1) can sense such NaCl disturbance [39]. In the same vein, the loss-of-function Arabidopsis fer mutants (*fer-4*) exhibit strong reduction in the late-stage of Ca^2+^ spikes compared to wildtype. This FER-dependent late-induction of Ca^2+^ activates cell wall reinforcement and maintains its integrity during growth recovery [39]. The essential role of RLKs FERONIA in salt adaptation is unquestionable. However, the late increase in cytosolic Ca^2+^ downstream of FER-dependent signals under salt stress makes one wonder about the potential role of FERONIA as an ionic sensor, particularly with knowing that rapid apoplastic alkalinization takes place within seconds under salinity (see Section 3.2) [40]. The extracellular alkalinization that is triggered by secreted peptide RALF (rapid alkalinization factor) regulates cell expansion in response to different developmental and stress stimuli [41,42]. Notably, RALF inhibits cell expansion of Arabidopsis primary root via interacting with FERONIA, and subsequently deactivates the PM H^+^-ATPase, resulting in apoplastic alkalinization [42]. Further research is still needed to clarify whether FERONIA acts downstream of RALF in response to salinity, as well as elucidating its up- and downstream signals.

Interestingly, by utilizing forward genetic screening, Jiang et al. [43] successfully isolated an Arabidopsis mutant, monocation-induced [Ca^2+^]i increases 1 (*moca1*), that is defective in salt-induced Ca^2+^ spikes. The authors identified MOCA1 as a glucuronosyltransferase for glycosyl inositol phosphorylceramide (GIPC) sphingolipids located on the plasma membrane. The authors also showed that MOCA1 is essential for NaCl-triggering depolarization of the cell-surface potential. In essence, Na^+^ binds to GIPCs, and facilitates Ca^2+^ influx that ultimately leads to Ca^2+^ spikes and activation of the downstream signals (Figure 1 [1^I^] and [2^C→1^]; see Section 3.1; [43]). Although the type of channel that fluxes Ca^2+^ has not been yet identified, the depolarization-activated DA-NSCCs could be plausible candidates. Indeed, subsequent rapid influx of Na^+^ into the cortical cytoplasm of plant roots occurs through the voltage-insensitive VI-NSCCs (Figure 1 [2^I^]), thereby depolarizing the cytosolic surface of the PM (Figure 1 [3^N→1^]) [44]. Therefore, further activation of the DA-NSCCs is established, which in turn enhances Ca^2+^ (as well as Na^+^ but to lesser extent) influx, and in combination with VI-NSCCs and MSCCs, shapes its cytosolic signature (Figure 1 [1^I^, 2^I^, 2^O^, 3^N→1→1^]) [22]. Therefore, the fluxing activity of different types of NSCCs play a crucial role in stress adaptation mechanisms. The PM of salt-sensitive plants are suggested to be predominantly equipped with late-activated, weakly selective hyperpolarization-activated NSCCs (HA-NSCCs), while the PM of salt-tolerant plants may contain more active NSCCs (DA-NSCCs and VI-NSCCs) [45,46]. For example, moderately salt-tolerant *Vitis rupestris* cells exhibit vigorous and rapid Na^+^ influx under saline conditions compared to salt-sensitive *Vitis riparia* cells [40,47]. This transiently rapid influx of Na^+^ ions could partially inhibit the K^+^ outward rectifiers (KORs) to maintain cellular K^+^/Na^+^ homeostasis (Figure 1 [3^N→3^]) [48]. Furthermore, it may act as a cheap osmolyte to counter salt-imposed osmotic stress (Figure 1 [3^N→2^]) [49]. 

On the contrary, although salt-adapting plants can derive benefits from initially influxing ions, overaccumulated Na^+^ in the cytosol is detrimental [50]. Treatment of grapes or tobacco BY-2 cells and *Arabidopsis* roots with high concentrations of NaCl results in increased reactive oxygen species (ROS) accumulation, leading to programmed cell death (PCD) [40,51,52]. Furthermore, salt-induced membrane depolarization inhibits PM ATPase (Figure 1 [3^N→1→2^]), but promotes KORs that subsequently efflux K^+^ out of the cell (Figure 1 [3^N→1→3^]) [43,53]. When the latter depletion is irrevocable, cells launch the PCD process [54]. Salt-tolerant plants therefore have to block additional Na^+^ influx by rapidly deactivating NSCCs (notably VI-NSCCs and DA-NSCCs) and remove excessive cytosolic concentrations. To achieve this, cyclic adenosine monophosphate (cAMP) or cyclic guanosine monophosphate (cGMP), which are produced via the mechanosensitive membrane-located cyclase, is induced within seconds by salt and osmotic stress, which enhances plant adaptation under increased salinity, but not during osmotic stress, via inhibiting VI-NSCC activity (Figure 1 [4^O→2^]) [55,56]. This rapid ionic-induced cyclic nucleotide monophosphate (cNMP) can activate the cyclic nucleotide gated (nonselective) cation channels (CNGCs) for further Ca^2+^ influxes, but it deactivates KOR (Figure 1 [4^O→1^ & 4^O→3^]) [48]. Subsequent signals of Ca^2+^ act upstream of the salt overly sensitive (SOS) pathway, which removes Na^+^ from the cytosol (Figure 1 [2^C→6&9^]) [44,45]. In addition, *Oryza sativa* phospholipase C (OsPLC1) was shown to trigger Ca^2+^ induction under NaCl, and the latter signal is substantial for controlling Na^+^ accumulation in leaf blades, hence improving salt adaptation [57].

Moreover, a plant regulates Na^+^ distribution at the systematic level through the high affinity K^+^ transporters (HKTs), with only one representing the gene *AtHKT1;1* in the Arabidopsis genome [58]. The expression site of *AtHKT1;1*, as well as *Oryza sativa OsHKT1;5* in the vasculature in roots and shoots, allows the plant to protect leaves against salinity via uploading K^+^ instead of Na^+^ in the xylem vessels [59,60]. The plant utilizes the negative effect of Na^+^-induced membrane depolarization as an adaption strategy. The specific overexpression of the *AtHKT1;1* in the Arabidopsis mature root stele, and in the outer cells of the root in both Arabidopsis and rice, results in decreasing Na^+^ accumulation in the shoot and improved salinity tolerance [61,62]. Furthermore, the salinity sensitivity of durum wheat is attributed to its poor capability in excluding Na^+^ from the leaf when compared to the salt-tolerant wheat relative *Triticum monococcum*, which contains Nax2 (TmHKT1;5-A). Thus, introducing *TmHKT1;5-A* into the durum wheat cultivar Tamaroi was sufficient to significantly reduce Na^+^ contents in leaves while increasing grain yield by 25% under increased salinity compared to near-isogenic lines without Nax2 (TmHKT1;5-A) [63]. In contrast, both constitutive 35S-overexpression of *AtHKT1;1* and *athkt1* single mutant show Na^+^ hypersensitivity as more Na^+^ ions overaccumulate in shoots under NaCl stress [61,64]. However, in the presence of 3 mM Ca^2+^ concentration, (but not 1 mM), T-DNA insertion mutations in *AtHKT1* (*hkt1–1* and *hkt1–2*) result in reducing the intracellularly accumulated Na^+^ and enhancing salinity tolerance of *sos3-1* mutant seedlings [65,66]. Interestingly, the Ca^2+^-dependent calmodulin-binding transcription activator 6 (CAMTA6) regulates the spatial expression of *HKT1;1*, as the Arabidopsis *camta6* mutant showed restricted expression of *HKT1;1* in the radicles. This mutant also showed enhanced salt adaptation during germination, but older seedlings were salt-sensitive [67]. These together point to the importance of the Ca^2+^ signature in harmonizing Na^+^ distribution on both cellular (SOS pathway) and systematic (HKT pathway) levels (Figure 1 [2^C→6&9^]) [60]. 

## 3. Decoding Signals to Understandable Cellular Language

### 3.1. Ca^2+^ Ion: a Cellular Central "Signaling-Maker”

Calcium plays a central role in all kingdoms of life, and is involved in nearly all biological processes. Despite such universality, signals of Ca^2+^ gain specificity from their signature patterns that are shaped by a powerful Ca^2+^-buffering capacity [68]. These Ca^2+^ signals can be perceived by adaptor/ Ca^2+^-modulated proteins, and are further propagated by releasing them from membrane-enclosed organelles, especially vacuoles [69]. In terms of plant–water relations, Ca^2+^ has a dual but paradoxical function. Submicromolar Ca^2+^ concentrations can phosphorylate AQP SoPIP2;1 at Ser^274^ by a PM-associated protein kinase, resulting in fluxing water under nonstressful conditions [8,70]; the resting cells exhibit nanomolar concentrations of Ca^2+^ in the cytosol (~100–200 nM) [71]. In contrast, the opposite occurs under water stress as active AQPs lead to cell turgor loss and plasmolysis. Therefore, preventing, or at least decreasing, water loss is the top priority of plants. Intriguingly, Ca^2+^ signals seem to be in/directly involved in AQP regulating mechanisms. As mentioned above, effluxing water out of the cell via PM AQPs might generate an endogenous turgor-driven mechanical stimulus that leads to cytoskeletal reorganization and/or affects PM dynamicity, which in turn activates the PM MSCCs-mediated Ca^2+^ influx. Indeed, under a different type of mechanical stimuli, for example, Ca^2+^_cyt_ peaks within seconds, showing stimulus-specific signature patterns in Arabidopsis roots [72], and in mannitol-, sorbitol-, or salinity-stressed seedlings (Figure 1 [1^I^, 2^O^, 4^O^ & 3^N→1→1^]) [25,73]. These studies show that pretreatment with a bona fide Ca^2+^ channel blocker inhibits not only Ca^2+^_cyt_ induction, but also pH changes and extracellular ROS. A PM fluxing system thus first initiates Ca^2+^_cyt_ increases where its signal governs ROS and H^+^ accumulation [37,72]. Indeed, Ca^2+^ spikes are accompanied by changes in cytosolic pH, and both Ca^2+^ and pH signatures act in harmony [74]. Furthermore, Ca^2+^ application and cytoplasmic acidification strongly reduce the hydraulic conductivity of isolated vesicles from Beta vulgaris in a dose-dependent manner [75]. Similar results were obtained when micromolar Ca^2+^ concentrations and low pH reduced PM AQPs in Arabidopsis suspension cells (Figure 1 [2^C→1^]) [76]. It remains unclear whether the enhancement of Ca^2+^ under water-related stress works directly on AQPs by promoting their dephosphorylation, as shown in isolated vesicles, or indirectly by increasing cytosolic transient changes in pH and/or extracellular ROS (Figure 1 [2^C→2&3^]). Further research is required to advance our knowledge about the exact role of water-induced Ca^2+^ on AQP gating.

Under increased salinity, however, a plant is further affected by Na^+^ ions. Therefore, it has to deploy parallel mechanisms to (1) arrest the additional Na^+^ influx, (2) remove it from the place of action (cytosol), as well as (3) reduce its side effects. The first task can be partially accomplished via cAMP or cGMP, while the Ca^2+^-dependent SOS pathway seems to be a central hub for Na^+^ exclusion and sequestration [77]. To this end, the rapidly salt-induced Ca^2+^_cyt_ promotes the kinase activity of the Ser/Thr protein kinase SOS2 by two nonredundant Ca^2+^ binding proteins, SOS3 and SOS3 homolog SCaBP8 (also known as calcineurin B-like protein 10 CBL10; Figure 1 [2^C→6&10^]; for more details about Ca^2+^-dependent protein kinases, [78,79]). The SOS3 interacts with SOS2 at the FISL/NAF motif in the C-terminus, and because of the SOS3 myristoylation motif at the N terminus, the resultant SOS3/SOS2 complex is PM-localized [80,81,82,83]. SOS3/SOS2 subsequently phosphorylates the C-terminal autoinhibitory domain of the PM Na^+^/H^+^ antiporter SOS1, relieving its autoinhibition and hence unleashing its Na^+^-efflux activity (Figure 1 [2^C→6-Root^]) [84,85]. On the other hand, SCaBP8 interacts with, and recruits, SOS2 to activate an unknown Na^+^-sequestrating target at the tonoplast (Figure 1 [2^C→10→1^]) [86]. In fact, the vacuolar Na^+^, K^+^/H^+^ antiporter (NHX)-type exchangers NHX1 and 2 demonstrate equivalent Na^+^/H^+^ and K^+^/H^+^ exchange, which are essential for maintaining intracellular K^+^ and pH homeostasis, turgor regulation, and stomatal function [87,88]. Thus, it is evident that the tonoplast-localized NHXs are plausible candidates for SCaBP8/SOS2 phosphorylation [89], but this needs confirmation. The SCaBP8/SOS2 complex is also involved in fine-tuning Ca^2+^ signals upon salinity by initially interacting with and activating AtANN4 (a member of AtANNEXINs Ca^2+^-dependent membrane binding proteins) resulting in further Ca^2+^ influx. However, AtANN4 is lately phosphorylated and deactivated by SCaBP8/SOS2 [90]. Moreover, the vacuolar-localized AtCaM15 interacts with the AtNHX1 at the C-terminal hydrophilic region in a Ca^2+^- and pH-dependent manner, resulting in it modifying its cation selectivity [91].

Interestingly, as the SOS3 functions mainly in roots, plants deploy the preferentially-shoot expressed SCaBP8 to activate SOS1 in the shoot via recruiting the SOS2 to the PM (Figure 1 [2^C→6-Shoot^]). However, as the N-terminal myristoylation domain is missing, SCaBP8 localizes the PM by its N-terminal hydrophobic domain [92]. Moreover, the PM-bound SCaBP8/SOS2 complex is further stabilized, and the activity of SOS1 is enhanced by SOS2-dependent phosphorylation to its interacting SCaBP8 partner at the C-terminal Ser^237^ in a NaCl-dependent manner (Figure 1 [3^N→4-Shoot^]) [93]. Recent phosphoproteomic approaches in Arabidopsis roots revealed that the phosphorylation of SOS1 and NHX1 takes place within 45–120 min of salinity increase [11]. However, salt-stressed Arabidopsis suspension cells showed rapid changes within 5–15 min of phosphorylation activity of SOS1 compared to NHX2 (and to a lesser extend NHX1), which started after 15–60 min [10]. The fast response activity of SOS1 is further confirmed as the Na^+^ content inside salt-stressed *V. rupestris* cells is halted from 2–10 min, followed by a significant reduction [47]. These results highlight the activation priority of the SOS3/SOS2 pathway under increased salinity in root tissues before SCaBP8/SOS2 occurs in the shoots. Intriguingly, neither SOS1 nor NHX1 can bestow a salinity adaptation when genetically engineered, and only a few successful results have been obtained [45,94]. In other words, although restricting the cellular Na^+^ strategy via the SOS pathway is essential, it must be integrated with other resilience pathways at the same time to eliminate NaCl-related disturbances. For instance, a plant must restore the electrochemical potential across the PM and the tonoplast, controlling the powerful second messenger H^+^ (Figure 1 [2^C→2^ & 2^C→10→3^]). Interestingly, 14-3-3 proteins, the positive regulators of PM H^+^-ATPases, can also bind to SOS2 at the Ser^294^ residue under nonstressful conditions, resulting in a repression of its basal kinase activity. However, NaCl reduces this interaction, thereby activating the SOS pathway for salt adaptation [95]. The involvement of Ca^2+^-promoted pathways and their crosstalk with H^+^ will be discussed later in this article, along with the cellular levels of NaCl-promoted ROS that have to be fine-tuned and correctly integrated in the salinity-adaptive machinery. 

Moreover, Ca^2+^ signals are also self-regulated, whether by further releasing Ca^2+^ from or sequestrating Ca^2+^ into intracellular organelles and the apoplast [96]. Of particular interest are the large central vacuoles that harbor different types of Ca^2+^-channels (e.g., TPC1), Ca^2+^/H^+^ exchangers (CAXs), and the Ca^2+^ pumps (autoinhibited Ca^2+^-ATPases; ACA4 and ACA11), which represent the major internal Ca^2+^ stores in mature plant cells [97]. The Arabidopsis TPC1/SV channel is a perplexing player in Ca^2+^ signaling [69]. This TPC1/SV transmembrane comprises two Shaker-like six-transmembrane (6-TM) domains connected by a cytosolic loop that contains two EF-hand Ca^2+^ binding domains, in addition to a voltage sensor [98,99,100]. Thus, TPC1/SV is Ca^2+^_cyt_-activated in a voltage-dependent manner, but inhibited by increased luminal Ca^2+^ (Figure 1 [2^C→7^]). In contrast, the *fou2* mutant that carries a point mutation D454N of the TPC1 gene remains active under 100-fold accumulation of vacuolar Ca^2+^ [101,102]. Interestingly, *fou2* exhibits a higher jasmonic acid (JA background that strongly increases upon wounding, as well as increased resistance to the fungus *Botrytis cinerea* [103]. This pronounced JA was suggested as unlikely to be attributed to TPC1/SV-mediated Ca^2+^ in *fuo2* mesophyll cells, but rather due to its insensitivity to higher levels of luminal Ca^2+^ (via CAXs pathway, as discussed later) that enables K^+^ fluxes and cellular K^+^ homeostasis [101]. Furthermore, aequorin-expressing wild type, *tpc1-2* knockout, and *TPC1*-overexpressing plants exhibit no difference in their Ca^2+^ signature pattern, or a Ca^2+^-dependent response under different stress types, including NaCl and mannitol-derived osmotic stress [104]. Thus, it is speculated that other vacuolar transporters, but not TPC1, mediate wounding-induced Ca^2+^ release through the tonoplast, finally resulting in JA overaccumulation [101]. However, using Arabidopsis plants expressing the cytoplasmic Ca^2+^ sensor YCNano-65, Choi et al. [105] showed that only localized NaCl application to Arabidopsis roots was able to generate Ca^2+^ waves at rates up to ~400 μm/s, which decreased by ∼25-fold in speed in the *tpc1-2* mutant. The improvement in salinity adaptation in *TPC1*-overexpressing plants compared to wild types and *tpc1-2* mutants suggest that the Ca^2+^wave/(TPC1/SV) elicits a rapid and systemic root-to-shoot signaling response in plants [105]. It is worth noting that although *AtTPC1* is ubiquitously expressed, these salt-specific Ca^2+^ waves are primarily channeled in the cortical and endodermal layers, and require ROS-supporting signals that are generated via AtRBOHD in a Ca^2+^-dependent manner [105,106]. In contrast, SOS1 is preferentially expressed in xylem parenchyma cells of the roots and shoots, where *AtHKT1;1* and *OsHKT1;5* are also expressed [59,84]. Hence, Na^+^ routes are largely confined to the xylem vessels, unless partially unloaded and replaced by K^+^ ions via AtHKT1;1 and OsHKT1;5 [60]. Therefore, it seems that plants retain a Ca^2+^-discrete root-to-shoot route, thereby giving priority to the Ca^2+^ signal over Na^+^ and K^+^ ions arriving at distal parts under increased salinity [107]. On the other hand, it might help prevent the Na^+^ signal within the waves of Ca^2+^.

Plants have evolved different mechanisms of Ca^2+^_cyt_-sequestration/buffering that interplay with Ca^2+^_cyt_-propagation strategies to guarantee fine-tuning (on/off) of stimulus-induced Ca^2+^ signaling, thereby preventing cellular damage [108]. For example, elevated Ca^2+^_cyt_ can deactivate the PM CNGCs via its interaction with Ca^2+^/CaM in a negative feedback loop [109]. Of paramount importance are the low affinity, high capacity vacuolar Ca^2+^ exchangers (CAXs) that effectively sequestrate Ca^2+^ into the vacuole subsequently after the Ca^2+^_cyt_ burst. In *Arabidopsis thaliana*, CAX transporters comprise six members that are further subgrouped into type 1A (CAX1, 3, and 4) and type 1B (CAX2, 5, and 6) [97]. Of interest are CAX1 and its closest homolog CAX3, which differ in their regulation and transport capacity; here, CAX1 is the main Ca^2+^-transporter compared to the weak CAX3 transporter [110,111]. The starting point of the NaCl-induced Ca^2+^ wave at the roots draws attention to the pivotal roles of the CAXs in fine-tuning Ca^2+^-signal transduction and ion homeostasis under increased salinity [112]. In particular, *CAX3* is predominantly expressed in the roots, and the *cax3* mutant is saline-sensitive [108,113]. It seems that, under increasing salinity, *CAX3* accumulates less Ca^2+^ inside the root vacuoles, which might not be able to efficiently impair TPC1 activity. It is important to note that the SOS1 is also preferentially expressed in the epidermal cells at the root tip where SOS3 is very strongly expressed, while generally also detected in the roots [84,92]. In contrast, AtNHX1 showed the expression pattern in nearly all tissues throughout plant development [114], but was not expressed at the root tip [84]. One can assume that much less Na^+^ is also accumulated inside root vacuoles, as high levels of luminal Na^+^, similar to luminal Ca^2+^, impair TPC1 activity [101,104]. Therefore, the simultaneous effects of SOS1, SOS3, and CAX3 allows Ca^2+^-activated root TPC1 to generate salt-specific Ca^2+^ waves from root to shoot (Figure 1 [2^C→9→ root^]). Once arriving via the cortical/endodermal route, Ca^2+^ signals prime the expressions of different NaCl-related genes in leaves, including JA-related genes [105]. However, JA is a dangerous switch, and sustained Ca^2+^ signals lead to harmful consequences [45,115]. Therefore, at this time, CAX1, an efficient Ca^2+^-transporter, responds. The CAX1 is the only member of the CAX genes that is highly expressed in Arabidopsis leaves, and, within the leaf, CAX1 accumulates at a 6-fold rate of the Ca^2+^ level in the mesophyll cell vacuoles compared to the epidermal cell vacuoles [110]. Interestingly, CAX1 and CAX3 can physically interact, forming a heteromeric CAX1–CAX3 protein complex with distinct functions when expressed in yeast and plant cells [116]. For instance, coexpression of both *CAX1* and *CAX3* in the yeast wild type enhances salt accumulation, while suppressing the salinity sensitivity of *nhx1* yeast strains, compared to their single expressions. The authors speculated that CAX3 might occasionally act as a cofactor when interacting with CAX1, modifying transport activity under stress or hormonal treatment [116]. Intriguingly, this could be the case when ABA upregulates the expression of CAX3 in guard cells, where the expression of *CAX1* is also detected [117]. Notably, CAX1 can also be activated via SOS2, which interacts with its hydrophilic N-terminal tail, although it remains unclear whether this interaction requires Ca^2+^ or not [118]. The high levels of Ca^2+^ at the luminal side are enough to inhibit the activity of TPC1 in the mesophyll cells (Figure 1 [ 2^C→10→2→ mesophyll^]) [98,99]. In addition, the arrival of Na^+^ ions to the shoot results in the accumulation of more Na^+^ ions inside the vacuoles of mature leaves via the SOS2/NHXs pathway, which further blocks TPC1 (Figure 1 [ 2^C→10→1→ mesophyll^]) [101]. This might explain why mesophyll cells or leaf discs derived from Arabidopsis wild type, *tpc1-2* knockout, or *TPC1*-overexpressing plants fail to exhibit differences in their Ca^2+^ signature patterns under NaCl stress [101,104]. Furthermore, when reaching intolerable levels, a plant drops its high Na^+^-containing old leaves to protect young leaves, thereby rescuing the whole plant from salt toxicity [49]. For complexity, the pattern of Ca^2+^ storage in cereal monocots (barley, wheat, and Sorghum bicolor) is shifted into leaf epidermal cells instead of mesophyll cells [119]. 

In summary, Ca^2+^ is a central signal maker at the cellular level across the whole plant. Ca^2+^-signals are highly intricate and are involved not only in regulating Na^+^, but also with other crucial secondary messengers (H^+^ and ROS), as well as ABA and JA. In addition, Ca^2+^-signals are self-regulated to guarantee the specificity of action, and to prevent Ca^2+^-induced cell death. Future research is required to explain more about this master signal element in both dicots and monocots.

### 3.2. Proton (H^+^): the Power of Cellular Buffering

The proton is a powerful, multifaceted cellular plant component involved in growth and developmental processes [120,121]. Moreover, rapid (within seconds) H^+^ entry takes place downstream of Ca^2+^ signals, probably through PM nonselective cation/anion channels, and is suggested to act as a second messenger under stress (Figure 1 [2^C→2^]) [45,72,122,123]. Therefore, plants have evolved different strategies to utilize it in a timely manner, and at the same time prevent its undesirable influences. For instance, the proton-consuming system is a metabolic-based fine-tuning mechanism that counteracts protonogenic reactions to strictly control the intracellular pH [124]. Furthermore, plant membranes are armed by different types of proton pumps that generate proton-derived pH gradients and, as a result, energize membranes with the required driving force for ion and metabolite transport [123]. Of particular interest are the PM electrogenic proton pumps (P-type H^+^-ATPases) that act coordinately with the vacuolar V-type H^+^-ATPases (V-ATPases) and V-pyrophosphatases (V-PPases) to extrude H^+^ out of the cytosol [125,126]. Under nonstressful conditions, PM H^+^-ATPases (12 members of the *Arabidopsis* PM H^+^-ATPases, AHAs) acidify the apoplast, promoting cell growth [127]. However, the opposite occurs under stress conditions, during which plants undergo cellular disturbances. For example, the biosynthetic capacity of the cellular currency, ATP, via oxidative phosphorylation becomes limited under flooding-triggered hypoxia/anoxia. Subsequently, PM H^+^-ATPases turn inactive and the resultant intracellularly accumulated H^+^ acidifies the cytosol [128]. Moreover, hypoxia-induced intracellular H^+^ acts as a second messenger and is sensed by the highly-conserved histidine residue in loop D of PIPs (corresponding to His^193^ in spinach SoPIP2;1), resulting in protonation-induced PIP gating (Figure 1 [5^H→1^]) [7,129]. Indeed, cytosol acidosis strongly reduces the hydraulic conductivity of isolated vesicles from Beta vulgaris and Arabidopsis suspension cells [75,76]. It is worth noting that, while PM H^+^-ATPases can also be deactivated by different types of stress, the H^+^-promoting PIP protonation is flooding-exclusive, and there are no reports regarding other stresses. 

Under high external pH values, the SOS2-like protein kinase PKS5 (CIPK11; SnRK3.22) was shown to negatively regulate the activity of AHA2 in a Ca^2+^ (SCaBP1/CBL2)-dependent manner. In contrast to Thr^947^ phosphorylation in the C-terminal regulatory domain of AHA2 that enhances interaction with, and subsequent activation by, 14-3-3 proteins, PKS5-induced phosphorylation of the Ser^931^ residue prevents interaction with 14-3-3 proteins [130,131]. Therefore, adaptation to high external pH was improved in the *pks5* mutant, and the membrane potential was not affected compared to the Arabidopsis wild type. Intriguingly, neither NaCl, which promotes membrane depolarization (similar to alkali stress), nor drought, which slowly steeps down the membrane potential, resulted in a phenotypic response when applied to the *pks5* mutant [130]. The upregulation of *PKS5* transcripts by NaCl, drought, or mannitol/glucose-induced osmotic stress suggest the involvement of other functionally redundant proteins with PKS5 under such conditions [130]. However, these stresses were applied at very high doses, especially NaCl, and it is possible that this induction of *PKS5* expression occurs in the cell death program, rather than the adaptation process. Undoubtedly, cellular accumulation of NaCl inhibits PM ATPase via membrane depolarization (Figure 1 [3^N→1→2^]). However, elevated salinity tolerance in halophyte quinoa, saltbush, and salt-tolerant barley genotypes were attributed to prompt activation of PM H^+^-ATPase, rather than higher transcription of AHAs, as observed in salt-sensitive Arabidopsis under increased salinity [132,133]. Here, Ca^2+^ signals activate PM H^+^-ATPase, as well as SOS2, by binding to the 14-3-3 proteins promoting its interaction with PKS5, and subsequently repressing its kinase activity (Figure 1 [2^C→4^]) [134]. Moreover, overexpressing the mutated version of PM H^+^-ATPase4 that lacks the autoinhibitory domain (∆PMA4) improved the salinity tolerance of transgenic tobacco [54,135]. Additionally, kinase activity of PKS5 can be repressed by a physical interaction with the chaperone J3, unleashing the PM H^+^-ATPase activity compared to *j3* mutants that also exhibit hypersensitivity to alkali and salt stress [136]. On the other hand, ABA enhances PKS5 kinase activity, promoting its interaction with, and phosphorylation of, ABA-insensitive5 (ABI5) at the Ser^42^ residue [137]. Interestingly, intracellular pH homeostasis has been shown to play a critical role in regulating the expression of ABA-biosynthetic genes, in general, and under PEG-induced osmotic stress, in particular [138]. Therefore, other possible mechanisms need to be investigated. 

Notably, post-translation regulation of PM H^+^-ATPases seems to be very intricate, since the regulatory/autoinhibitory C-terminal domain can be phosphorylated at different sites, resulting in inactive/active forms of AHAs [131]. Phosphoproteomic approaches have shown that both AHA1 (AT2G18960.1; known also as open stomata2 OST2) and AHA2 (AT4G30190.1) were rapidly phosphorylated within 5 min and 15 min, respectively, in Arabidopsis suspension cells under saline conditions [10]. However, the phosphorylation of AHA1 and AHA2 was shown to occur at Thr^948^ (corresponding to Thr^947^ in AHA2) and Ser^899^, respectively, resulting in the activation of the predominantly shoot expressed AHA1, but the inactivation of the mainly root-expressed AHA2 [10]. It seems that there is an interaction within AHAs, and with other PM transporters, such as SOS1, that become gradually phosphorylated in roots [11], but that peak at 15 min in suspension cells under exposure to salinity [10]. In fact, *V. rupestris* suspension cells exhibited a reduction of extracellular alkalinization (H^+^ efflux) concomitant with decreasing cellular levels of Na^+^ after 10–15 min of salinity exposure [45,47]. In addition, constitutively active AHA1 in Arabidopsis (*ost2* mutant) results in stomatal closure failure and an ABA-insensitive phenotype [139,140,141]. However, phosphorylation of AHA1 and AHA2 in the Ser^2^ residue opens new questions [11]; further research is required to enhance our understanding in this regard. 

The activity of important transporters, such as NHXs and CAXs, on the tonoplast are affected by the efficiency of V-ATPases and V-PPases, and vice versa [126,142]. For example, *cax1* and *cax3* mutations decrease V-ATPase activity, maybe due to the accumulation of luminal H^+^ [113,143]. In addition, Ca^2+^ signaling is also interconnected to V-ATPase via the SOS pathway. Here, the Ca^2+^-dependent SCaBP8/SOS2 can activate V-ATPase by interacting with its regulatory subunits VHA-B1 and 2, resulting in fine-tuning of Ca^2+^ signaling and ion homeostasis under salinity [69,118,144]. In addition, in salt-stressed *Solanum lycopersicum*, the SCaBP8/SOS2 complex is also proposed to activate the tonoplastic H^+^-pyrophosphatase (SlAVP1) and SlV-ATPase, as well as SlTPC1, resulting in the regulation of Na^+^ and Ca^2+^ fluxes in the leaf vacuole, and hence improving salt adaptation (Figure 1 [2^C→10→4^]) [145]. Subsequently, stimulated V-ATPases reduce the luminal pH, which, in turn, promotes the interaction of AtCaM15 with the C-terminal hydrophilic region of AtNHX1, resulting in decreasing Na^+^/H^+^ exchange activity in yeast vacuoles [91]. Although the AtCaM15/AtNHX1 interaction was shown to take place in plants, modification of NHX activity has not been proven yet. However, if this was the case, it might be a mechanism to initially prevent Na^+^ accumulation in leaf vacuoles, thereby favoring Na^+^ redistribution. Interestingly, 14-3-3 proteins, the positive regulators of PM H^+^-ATPases, can also bind to the SOS2 junction domain where a critical Ser^294^ phosphorylation site exists, resulting in kinase activity repression under nonstressful conditions, but not under salinity exposure [95]. Therefore, it seems that plants pay critical attention to the Ca^2+^-activated SOS pathway to further fine tune Ca^2+^-signals. 

### 3.3. ROS: Do Not Completely Unleash the Fire

ROS are inevitable byproducts of plant aerobic metabolism that turn toxic, unless ameliorating measures are taken. Interestingly, plants have not only evolved indispensable ROS-scavenging strategies, but also deliberately generate ROS as pivotal cellular second messengers [146]. Therefore, plants master a delicate fine-tuning to channel ROS in desirable processes. As discussed earlier, the within-second stimulus-induced elevations of Ca^2+^_cyt_ triggers the accumulation of H^+^ in the cytosol, as well as ROS [72,73]. The latter signal is launched when Ca^2+^_cyt_ activates the PM NADPH oxidase ROBHD via CPK5, resulting in superoxide O_2_^•−^ production in the apoplast, which is converted to H_2_O_2_ by superoxide dismutase [147]. These ROS signals act locally and systemically under different stress types, including salinity, as well as in cooperation with other cellular signals, especially, Ca^2+^ [148]. For instance, ROS can positively feedback on the Ca^2+^ signal, activating the hyperpolarization NSCCs [149]. Many excellent reviews on ROS signaling have been published recently [150,151,152]. One of the intriguing questions that often rises in that regard is how such elevated extracellular ROS are sensed on the cell surface. Recently, by utilizing forward genetic screens in combination with Ca^2+^ imaging, Wu et al. [153] identified an Arabidopsis mutant that exhibits low Ca^2+^ influx upon H_2_O_2_ treatment; hence came its name hydrogen-peroxide-induced Ca^2+^ increases (*hpca*). HPCA1 belongs to the leucine-rich-repeat (LRR) receptor kinases, and was shown to mediate stomatal closure via the activation of Ca^2+^ channels in guard cells under H_2_O_2_ application [153]. Further research is required to elucidate the role of HPCA1 in response to developmental and environmental stimuli. 

Under water-related stress, reserving cellular water is among the top priorities of the cell and is achieved via controlling AQP (PIP) function. The Ca^2+^-triggered H_2_O_2_ on the apoplast can rapidly diffuse into the cell via PM PIPs [154], and thereby might feed negatively on PIPs by promoting their internalization. In Arabidopsis roots, H_2_O_2_-induced PIP internalization under salinity stress, as well as H_2_O_2_ per se, can be removed by catalase application [155]. Moreover, H_2_O_2_ has been shown to dephosphorylate AtPIP2;1 at the Ser^283^ residue, similar to salinity exposure, but within a shorter application time, and hence, the nonphosphorylated forms of AtPIP2;1 accumulate intracellularly (Figure 1 [6^R→1^]) [12]. Therefore, downregulation of root hydraulic conductivity under salinity is attributed to the ROS-activated cell signal chain where Ca^2+^-signaling is partially involved, rather than direct oxidative action [155]. Interestingly, H_2_O_2_ stress resulted in phosphorylating AHA1 and AHA2 at the same sites, Thr^948^ and Ser^899^, respectively, similar to salinity stress, but with a different kinetic pattern [10]. These results indicate the interconnection of ROS and H^+^ signals, adding additional levels of complexity to cellular signals. In addition, the role of ROS in activating ABA and JA signals adds further layers of complexity (Figure 1 [6^R→2&3^]) [151].

On the other hand, overaccumulating ROS under pathogen stress [156] acts as a fire that burns the local infected site with a PCD strategy to restrict the spread of invaders. In the context of abiotic stress, PCD is unlikely to be a suitable response in plants [47,52]. Therefore, plants have evolved ROS-homeostatic strategies for the timely scavenging of ROS. To do so, gene expression of the ROS-scavenging enzymes peroxidase (POD), Glutathione S-transferase (GST), and catalase (CAT) were enhanced in cotton *Gossypium davidsonii* leaves and roots (Figure 1 [6^R→antioxidant system^]) [157]. Interesting, Ca^2+^ and its related signals seem to play a crucial role in preventing ROS-induced cellular damage. For example, *Oryza sativa OsCPK4* expression was promoted in rice roots under salt and drought stresses, as well as ABA treatment, and *OsCPK4* transgenic rice plants are salt- and drought-tolerant. The upregulation of redox-finetuning transcripts, such as *POD*, *GST*, and *Laccase* in the *OsCPK4* transgenic lines, is evidence that OsCPK4 is involved in protecting cellular membranes from ROS-induced oxidative damage [78,158,159]. Future research shall further unravel additional layers of the intricate networks among ROS and other signals, such as Ca^2+^ and H^+^.

## 4. Conclusions

To understand the underlying mechanisms of water-related stress, it is important to contextualize cellular events. The current review summarizes new advances in the field that might help in formulating an integrative perspective of cellular events under salt-related stress. It is also of paramount interest that future research focuses on the puzzling points, in addition to running parallel studies that investigate the phenomenon at different plant levels. In addition, comprehensive parallel studies on tolerant and sensitive cultivars will aid in correlating signals/events to the right cellular context.

## Abbreviations

AQPs	Aquaporins
PIPs	Plasma membrane intrinsic proteins
TIPs	Tonoplast intrinsic proteins
PM	Plasma membrane
MSCCs	Mechanosensitive – NSCCs
OST1	Open stomata 1
ABA	Abscisic acid
NSCCs	Nonselective cation channels
KORs	K^+^ outward rectifiers
Ψs	Osmotic potential
MCA	Mid1-Complementing Activity
OSCAs	reduced hyperosmolality-induced [Ca2+]i increase
RLKs	receptor-like kinases
MTs	microtubules
FER	the malectin-like-domain RK FERONIA
RALF	rapid alkalinization factor
MOCA1	a glucuronosyltransferase for glycosyl inositol phosphorylceramide (GIPC) sphingolipids
GIPC	Glycosyl Inositol Phospho Ceramides
DA-NSCCs	Depolarization-activated-NSCCs
VI-NSCCs	Voltage-insensitive- NSCCs
HA-NSCCs	hyperpolarization-activated NSCCs
ROS	Reactive oxygen species
PCD	Programed Cell Death
cAMP	cyclic adenosine monophosphate
cGMP	Cyclic guanosine monophosphate
cNMP	ionic-induced cyclic nucleotide monophosphate
CNGCs	cyclic nucleotide gated NSCCs
SOS	Salt overly sensitive
PLC	phospholipase C
HKTs	high affinity K+ transporters
CAMTA6	Ca^2+^-dependent calmodulin-binding transcription activator 6
SCaBP8	SOS3-like calcium binding protein 8
	(also known as calcineurin B-like protein 10, CBL10)
NHXs	Vacuolar Na+/H+ exchangers
AtANN4	a member of AtANNEXINs calcium-dependent membrane binding proteins
TPC1	Two pore channel 1
CAXs	Ca^2+^/H^+^ exchangers
JA	Jasmonic acid
JA-Il	Jasmonyl isoleucine
RBOHD	Respiratory burst oxidase homolog D
H^+^-ATPases	PM electrogenic proton pumps
V-ATPases	vacuolar V-type H^+^-ATPases
V-PPases	vacuolar V-pyrophosphatases
PKS5	SOS2-like protein kinase
ABI5	ABA-insensitive5
CPKs	calcium-dependent protein kinases
CAT	Catalase
POD	Peroxidase
GST	Glutathione S-transferase
